# Physiological and biochemical responses of wheat to synergistic effects of selenium nanoparticles and elevated CO_2_ conditions

**DOI:** 10.3389/fpls.2023.1183185

**Published:** 2023-07-13

**Authors:** Emad A. Alsherif, Dina Hajjar, Mohammad Aldilami, Hamada AbdElgawad

**Affiliations:** ^1^ Biology Department, College of Science and Arts at Khulis, University of Jeddah, Jeddah, Saudi Arabia; ^2^ Department of Biochemistry, College of Science, University of Jeddah, Jeddah, Saudi Arabia; ^3^ Integrated Molecular Plant Physiology Research, Department of Biology, University of Antwerp, Antwerp, Belgium

**Keywords:** antioxidant, elevated CO_2_, metabolite, selenium nanoparticles, wheat

## Abstract

Elevating CO_2_ (eCO_2_) levels will change behavior and the effect of soil fertilizers and nutrients. Selenium NPs (SeNPs) have arisen as an alternative to conventional Se fertilizers to enrich crops. However, it remains unclear whether eCO_2_ will change the biological effects of soil SeNPs on plant growth and metabolism. The current study aimed to shed new light on the interactive impacts of eCO_2_ and SeNPs on wheat plants. Accordingly, the attempts were to reveal whether the application of SeNPs can modulate the eCO_2_ effects on wheat (*Triticum aestivum* L.) physiological and biochemical traits. With this goal, a pot experiment was carried out where the seeds were primed with SeNPs and plants were grown under two levels of CO_2_ concentrations (ambient CO_2_ (aCO_2_, 410 μmol CO_2_ mol^−1^; and eCO_2_ (710 μmol CO_2_ mol^−1^)) during six weeks after sowing. Although SeNPs+eCO_2_ treatment resulted in the highest accumulation of photosynthetic pigment content in leaves (+49-118% higher than control), strong evidence of the positive impacts on Rubisco activity (~+23%), and stomatal conductance (~+37%) was observed only under eCO_2_, which resulted in an improvement in photosynthesis capacity (+42%). When photosynthesis parameters were stimulated with eCO_2_, a significant improvement in dry matter production was detected, in particular under SeNPs+eCO_2_ which was 1.8 times higher than control under aCO_2_. The highest content of antioxidant enzymes, molecules, and metabolites was also recorded in SeNPs+eCO_2_, which might be associated with the nearly 50% increase in sodium content in shoots at the same treatment. Taken together, this is the first research documenting the effective synergistic impacts of eCO_2_ and SeNPs on the mentioned metabolites, antioxidants, and some photosynthetic parameters, an advantageous consequence that was not recorded in the individual application of these treatments, at least not as broadly as with the combined treatment.

## 1 Introduction

Carbon dioxide (CO_2_) emissions are gradually increasing due to expanding industrial activities and fossil fuel use, conversion of natural ecosystems, and poor agricultural and forestry management, from 280 μmol CO_2_ mol^–1^ in pre-industrial times to the current 416 μmol CO_2_ mol^–1^ and more than 700 μmol CO_2_ mol^–1^ by the end of this century (Fang et al., 2022; Shabbaj et al., 2022). This CO_2_ rise is the primary cause of climate change and global warming, resulting in increasing severity and iteration of climate-inordinate phenomena such as flooding, environmental stress (e.g., salinity, drought, high temperature, etc.), forest fires, heavy rain, and heat waves (AbdElgawad et al., 2014). As a result, elevated CO_2_ (eCO_2_) is increasingly recognized as a main factor affecting global crop production, because an increase in primary carbon sources influences crop growth, biomass production, the photosynthesis cycle, and metabolite profiling differently and significantly, even in non-stressed plants (Jing et al., 2016; Kaur et al., 2023). In recent years, there has been an increasing amount of literature on the responses of wheat plants to elevated atmospheric CO_2_ through improving photosynthesis capacity, declining transpiration and water use, and resulting in a productive advantage and higher grain yield (Ainsworth and Rogers, 2007). Accordingly, previous research has reported analyses of field experiments on wheat in different growing areas that demonstrated the increment in grain yield and yield components at high CO_2_ levels under non-environmental stress conditions, suggesting more boost in wheat grain number per unit surface area than the increase in grain weight (Wang et al., 2013; Broberg et al., 2019; Blandino et al., 2020). Likewise, data from several sources have identified the increased wheat productive response to elevated CO_2_ is associated with physiological mechanisms (Wang et al., 2013; Dubey et al., 2015), especially synthesizing bioactive compounds and antioxidant metabolites in non-stressed wheat plants (Blandino et al., 2020). Sreeharsha et al. (2019) considered the carbon supply under eCO_2_ as a factor for better plant growth because of its affecting sink capacity and mediating better N assimilation. Improved photosynthesis capacity and photosystem-II efficiency under eCO_2_ conditions have also reported through increasing chlorophyll a fluorescence characteristics (Sekhar et al., 2015).

Selenium (Se) deficiency, as an essential trace element for human health, is one of the foremost concerns in the rising risks of numerous illnesses and cancers (Liu et al., 2021). The nutritional deficit in staple foods is typically caused by plant production in mineral -poor soils, resulting in nearly two billion people worldwide suffering from nutritional deficiencies (Lanza and dos Reis, 2021). Agronomic biofortification is an agricultural technique employed to enrich crops with target nutrients, such as selenium, to ultimately improve crop-based food quality (Reis et al., 2017). Since the high mobility and availability of Se conventional chemical fertilizers have become a major concern in environmental pollution (Rosenfeld et al., 2017), there has been a significant increase in the potential applications of nanotechnology in Se supplements with significantly higher efficiency and lower toxicity (Liu et al., 2021). Selenium nanoparticles (SeNPs) have recently gained prominence due to their beneficial effects on plant growth, photosynthesis, and metabolite biosynthesis, as well as their association with plant oxidative stress tolerance (Lanza and dos Reis, 2021). Application of SeNPs as foliar (Ikram et al., 2020), seed priming (Nawaz et al., 2013), soil treated (Lyu et al., 2022) and under hydroponic conditions (Hu et al., 2018) resulted in improved nutritional values and physiological traits of wheat plants under both stress and non-stress conditions. Moreover, it has already been reported that eCO_2_ can increase the allocation of photosynthates to the roots (Habeeb et al., 2020). Also, it has been suggested that root exudates and CO_2_ produced via root respiration can acidify the rhizosphere, consequently making some nutrients more available to plants (Duval et al., 2012). Thus, eCO_2_ could change the behaviour of soil nutrients and thus modify their availability and biological impact on plants (Du et al., 2017). Similarly, eCO_2_ improved the potential for roots to uptake more nutrients such as Se, from the soil (Du et al., 2023), which consequently led to further growth and development in plants under eCO_2_.

SeNPs have arisen as an alternative to conventional Se fertilizers to enrich crops.

Although much research has been carried out on eCO_2_ and NPs, it remains unclear whether eCO_2_ will change the biological effects of soil SeNPs on plant growth and metabolism. It has already been reported that diverse metabolic processes can be modified under eCO_2_ (Mishra et al., 2013; AbdElgawad et al., 2016; Du et al., 2023). In Despite the research summarized above, the present study aimed to determinehow eCO_2_ can affect the biological impact of SeNPs on the biochemical and physiological shifts in eCO_2_-treated plants. We hypothesized that the application of SeNPs could synergistically and positively affect plant growth, photosynthesis parameters, and biochemistry in eCO_2_-treated wheat plants, as compared to those in control plants grown under ambient CO_2_ conditions.

## 2 Materials and methods

### 2.1 Experimental setup

Eight healthy wheat seeds (var. Giza 119) were sterilized by drenching in sodium hypochlorite solution (1% v/v) for 10 min and were planted in potting mix (Tref EGO substrates, Moerdijk, The Netherlands) in pots (15 cm diameter × 15 cm height) filled with a mixture (1:1, v/v) of loamy soil and organic compost at a humidity of 300 mg water g^–1^ dry soil. Plants were grown for six weeks after planting at controlled-environment reach-in growth chambers with a constant regime of 20°C air temperature, 14/10 h day/night photoperiod, 150 μmol m^−2^ s^−1^ photosynthetically active radiation until the tillering stage and then 550 μmol m^−2^ s^−1^ until jointing stage, and ~65% soil water content.

Three replications and a fully randomized design were used in the experiment, which contained four treatments, including two levels of selenium nanoparticles (SeNPs and control), and two levels of air CO_2_ concentrations (ambient CO_2_ (aCO_2_, 410 μmol CO_2_ mol^−1^; and elevated CO_2_ (eCO_2_, 710 μmol CO_2_ mol^−1^)). To apply the SeNPs treatment, the prepared seeds were treated in a suspension with 25 mg L^-1^ of SeNPs (purity of 99.99%; density of 4.79 g cm^−3^; American Elements, Los Angeles, CA, USA) at room temperature for 10 h in an ongoing shaking (IKA KS 501 shaker, Staufen, Germany), and washed thrice with distilled water. Soils were also mixed with 200 mL of 25 mg L^-1^ of SeNPs, however the control soils were mixed with 200 mL of distilled water. The rough aggregates in the aqueous suspension of SeNPs were sidestepped by sonication (Selim et al., 2022). The morphological characteristics of the SeNPs structure were also validated using a scanning electron microscope (SEM manufactured by JEOL JSM-6510, LA, Japan). The applied dose of Se-NPs was recommended in the previous experiments examining the effect of various concentrations of SeNPs (0, 10, 25, 50, and 75 mg L^−1^) on plants (Albqmi et al., 2023). SeNPs concentration was chosen based on the results of preliminary assay, in which the impact of target SeNPs dose induce 25% induction in plant dry weight. The CO_2_ was also emitted into the airflow of the chamber and was maintained at the target CO_2_ level using a CO_2_ analyzer (WMA-4, PP Systems, Hitchin, UK). Seeds used for control treatments (aCO_2_ and eCO_2_) were treated with distilled water for 10 h.

### 2.2 Sampling

#### 2.2.1 Plant biomass

Plant shoot tissues were harvested six weeks after planting, and a part of them was used to measure the plant’s fresh and dry matter, and the rest for following physiological and biochemical analysis.

#### 2.2.2 Determination of photosynthesis parameters

Chlorophyll and carotenoids in the leaves were measured spectrophotometrically based on the modified Porra (Porra, 2002) technique by reading the absorbance of extracted samples at the wavelengths of 665.2, 652.4 and 470 nm using a microplate reader (Synergy Mx, Biotek Instruments Inc., Vermont, VT, USA) (Yaghoubi Khanghahi et al., 2019). High-performance liquid chromatography (HPLC; Shimadzu, Hertogenbosch, The Netherlands) was used to measure β-carotene, zeaxanthin (Gilmore and Yamamoto, 1991), and lutein (Fratianni et al., 2015) in leaves, as fully described by previous studies.

The photosynthesis rate (P_N_) and stomatal conductance (gs) of the last developed leaves were measured using the LI-COR portable photosynthesis system (LI-COR 6400/XT, USA). The maximum efficiency of photosystem II in dark-adapted leaves (Fv/Fm) was also determined using a Pulse Amplitude Modulated fluorometer (PAM–2500, Walz, Germany), in which Fm and Fv are the maximum fluorescence and the variable fluorescence, respectively, as fully described by Genty et al. (1989). Briefly, the leaves were acclimated to the dark for 15 min using the dark leaf clips (DLC-8) to measure the basal and maximum fluorescence in response to low-intensity light (*<*0.1 *μ*mol photons m^−2^ s^−1^, red light) and a saturating light pulse (*>*8,000 *μ*mol photons m^−2^ s^−1^, white light) was turned on for 1 s (Yaghoubi Khanghahi et al., 2019).

Ribulose-1,5-biphosphate carboxylase: oxygenase (Rubisco) activity of leaves was determined spectrophotometrically based on the oxidation of nicotinamide adenine dinucleotide (NADH) at 340 nm (Lobo et al., 2015). Briefly, Rubisco activity was measured in as assay reaction mixture containing bicine (100 mM; pH 8.0), KHCO_3_ (25 mM), MgCl_2_ (20 mM), ATP (3.5 mM), phosphocreatine (5 mM), glyceraldehyde 3-phosphate dehydrogenase (80 nkat), 3-phosphoglyceric phosphokinase (80 nkat), creatine phosphokinase (80 nkat), and NADH (0.25 mM). The extract was incubated at 25 °C in the assay buffer for 15 min to complete the activation of Rubisco in the assay solution (Lobo et al., 2015).

#### 2.2.3 Determination of mineral content in plant and soil

Total-reflection X-ray fluorescence spectrometry (TXRF; Bruker Nano GmbH, Berlin, Germany) and an ion chromatograph (Shimadzu, Japan) were used to measure the concentration of selenium (Se), calcium (Ca), phosphorus (P), sodium (Na), potassium (K), magnesium (Mg), zinc (Zn) and sulfur (S) in plants and Se in soil (Yoshida et al., 2016; Bamrah et al., 2019).

#### 2.2.4 Assessment of antioxidant metabolites and enzymes

Samples were homogenized in 1 ml of buffer [50 mM potassium phosphate, pH 7.0, 10% (w/v) polyvinyl pyrrolidone (PVP), 0.25% (v/v) Triton X-100, 1 mM phenylmethylsulfonyl fluoride (PMSF), 1 mM ASC] and centrifuged to get a clear supernatant for measuring the activity of the antioxidant enzymes. Accordingly, superoxide dismutase (SOD) was measured based on the inhibition of nitroblue tetrazolium (NBT) reduction at 560 nm (Dhindsa et al., 1982). Peroxidase (POX) activity was determined according to the pyrogallol oxidation, as fully described by Kumar and Khan (1982). Catalase activity was assessed based on the breakdown of H_2_O_2_ at 240 nm (Aebi, 1984). The estimation of ascorbate peroxidase (APX) and glutathione reductase (GR) activities was fully explained by Murshed et al. (2008). Glutathione peroxidase (GPX) activity was determined based on the reduction in NADPH absorption at 340 nm (Drotar et al., 1985). Reduced ascorbate (ASC) content was assessed based on the method of Foyer et al. (1983).

Total antioxidant capacity (TAC) was measured through the ferric-reducing antioxidant power (FRAP) method by employing Trolox as a reference base (Selim et al., 2022). Briefly, the samples were extracted in ethanol (80% v/v) and centrifuged (at 14,000× *g* for 20 min at 4°C). The FRAP reagent was prepared by adding FeCl_3_ (20 mM) to the acetate buffer (0.25 M). The FRAP reagent (FeCl_3_ (20 mM) and acetate buffer (0.25 M)) was mixed with extracts, and the reading was measured at 593 nm. The quantification of flavonoids and polyphenols content was carried out using aluminum chloride calorimetric and Folin–Ciocalteu assays, based on the method of AbdElgawad et al. (2020) and Zhang et al. (2006), respectively. Tocopherol content was measured using HPLC, in which Dimethyl tocol (DMT; 5 ppm) was used as an internal standard, as fully described by AbdElgawad et al. (2015). The quantification of organic acids in soil extracted from the rhizosphere of wheat plants was done using HPLC (Lawongsa et al., 1987).

### 2.3 Statistical analysis

The SigmaPlot software package was used to perform all statistical calculations, including a two-way analysis of variance (ANOVA), the Tukey’s HSD (honestly significant difference) test, Pearson correlation, and graph creation. The results were expressed as the mean ± standard deviation. The significance level (α) for Tukey test and correlation analysis was set at 0.05.

## 3 Results

### 3.1 Photosynthetic parameters

**Figure 1:**
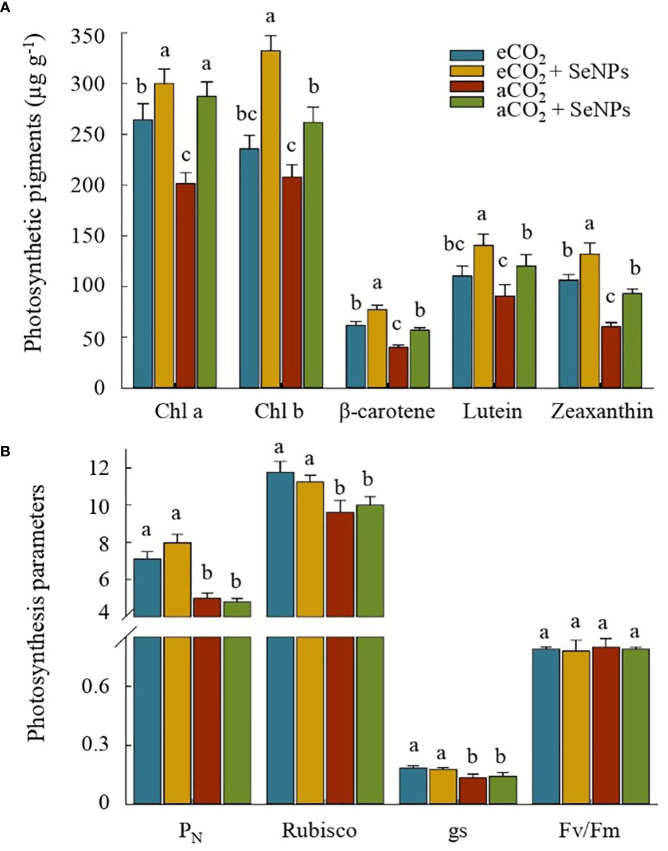
The effect of selenium nanoparticles (SeNPs) on the photosynthetic pigments **(A)** and photosynthesis parameters (µmol CO_2_ m^-2^ s^-1^ for P_N_. mmol CO_2_ m^-2^ s^-1^ for gs, and mg g^-1^ protein for Rubisco) **(B)** in plants under ambient CO_2_ (aCO_2_) and elevated CO_2_ (eCO_2_). Means in each parameter followed by similar letter(s) are not significantly different at 5% probability level (Tukey test). P_N_, Photosynthesis rate; gs, Stomatal conductance; Chl a, Chlorophyll a; Chl b, Chlorophyll b; F_v_/F_m_, maximum efficiency of PSII photochemistry in dark-adapted leaves.

The results of some photosynthetic parameters are presented in **Figure 1**. Accordingly, the highest concentrations of Chl a, Chl b, β-carotene, lutein, and zeaxanthin belonged to the selenium nanoparticle treatment in eCO_2_-treated plants (SeNPs+eCO_2_), which were about 13–41% and 49–118% higher than those in control plants (*P* < 0.05) under eCO_2_ and aCO_2_ conditions, respectively (**Figure 1A**). The improvement of the accumulation of pigments in response to the treatments led us to investigate their effect on some of the most important photosynthetic parameters of the plant, as shown in **Figure 1B**. Interestingly, for those treated with SeNPs, no changes were found in photosynthesis rate (P_N_), Rubisco activity, stomatal conductance (gs) and the maximum quantum yield of photosystem II (F_v_/F_m_) as compared to control plants under both eCO_2_ and aCO_2_ conditions (**Figure 1B**). Moreover, although, strong evidence of the positive impacts of elevated CO_2_ on P_N_, gs, and Rubisco activity were observed (*P* < 0.05), F_v_/F_m_ was not obviously affected by the treatments (*P* > 0.05).

### 3.2 Antioxidant enzymes and molecules

**Figure 2:**
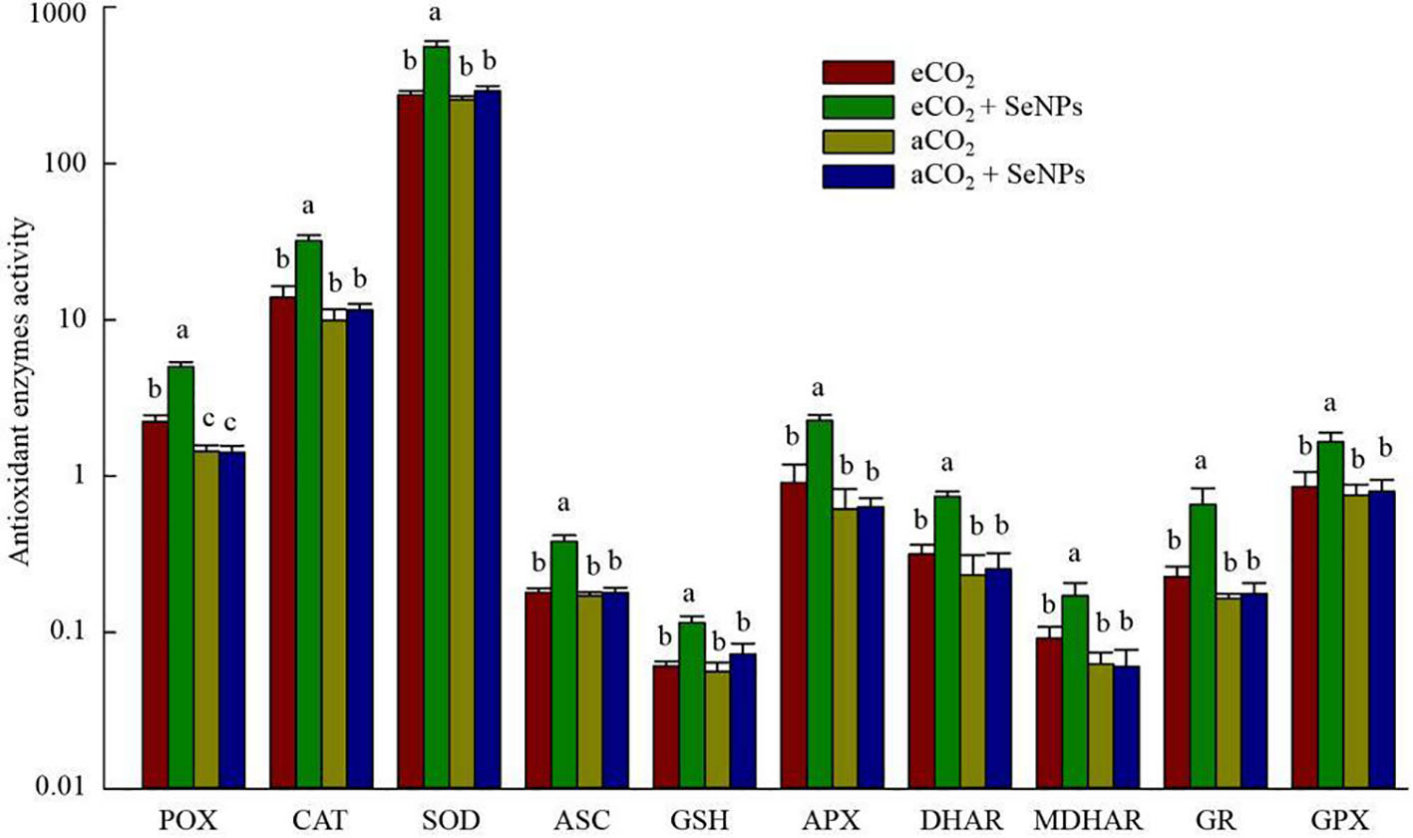
The effect of selenium nanoparticles (SeNPs) on the antioxidant enzymes (μmol min^-1^ mg^-1^ protein) and metabolites (µmol g^-1^ FW) in plants under ambient CO_2_ (aCO_2_) and elevated CO_2_ (eCO_2_). Means in each parameter followed by similar letter(s) are not significantly different at 5% probability level (Tukey test). GR, Glutathione reductase; GPX, Glutathione peroxidase; POX, Proxidase; CAT, Catalase; SOD, superoxide dismutase; DHAR, Dehydroascorbate reductase; MDHAR, Monodehydroascorbate reductase; ASC, Ascorbate; APX, Ascorbate peroxidase; GSH, Glutathione.

The potential of modifications in the antioxidant defense system was assessed by measuring the concentration of antioxidant enzymes and metabolites in plants in response to the treatments. The highest activity of POX, CAT, and SOD enzymes was found in the combined treatment (SeNPs+eCO_2_), in which the activity of these antioxidant direct-scavenging enzymes significantly (*P* < 0.05) increased up to 103-131% and 117-247% higher than the control plants under eCO_2_ and aCO_2_ conditions, respectively (**Figure 2**). Similarly, the individual applications of SeNPs and eCO_2_ treatments did not affect the biochemical components involved in the ascorbate-glutathione (ASC/GSH) pathways (*P* > 0.05). Accordingly, only the combined treatment (SeNPs+eCO_2_) caused a significant (*P* < 0.05) boost in the content of the antioxidant enzymes and metabolites, including ASC, GSH, APX, DHAR, MDHAR, GR, and GPX, by 90–190% higher than eCO_2_ and 105–298% greater than the aCO_2_ treatment (**Figure 2**).

**Figure 3:**
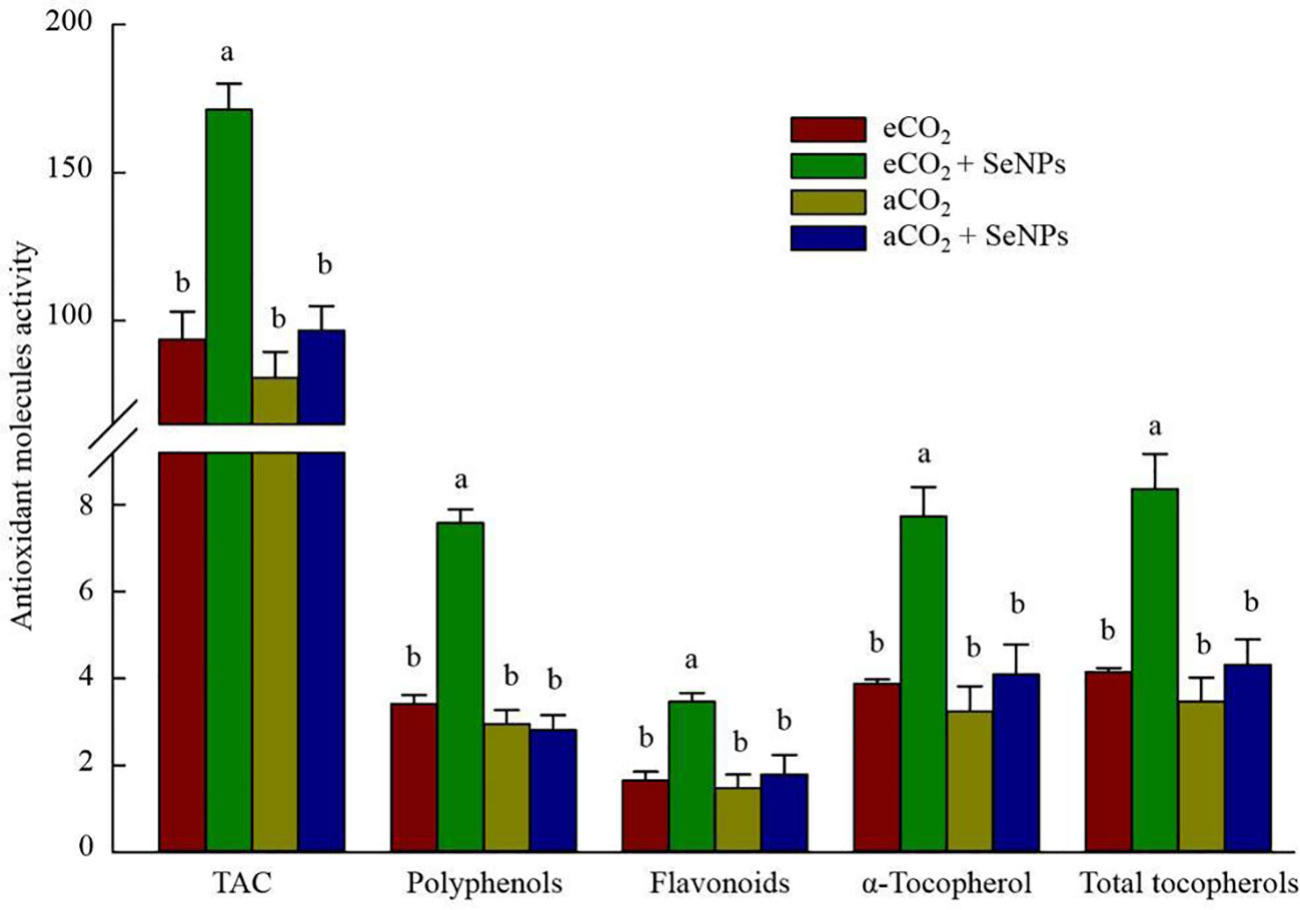
The effect of selenium nanoparticles (SeNPs) on the antioxidant molecules (µmol Torolex g^-1^ FW for TAC; mg GAE g^-1^ FW for polyphenols; mg Quercetin g^-1^ FW for flavonoids; ng g^-1^ for tocopherols) in plants under ambient CO_2_ (aCO_2_) and elevated CO_2_ (eCO_2_). Means in each parameter followed by similar letter(s) are not significantly different at 5% probability level (Tukey test). TAC, Total antioxidant capacity.

**Figure 4:**
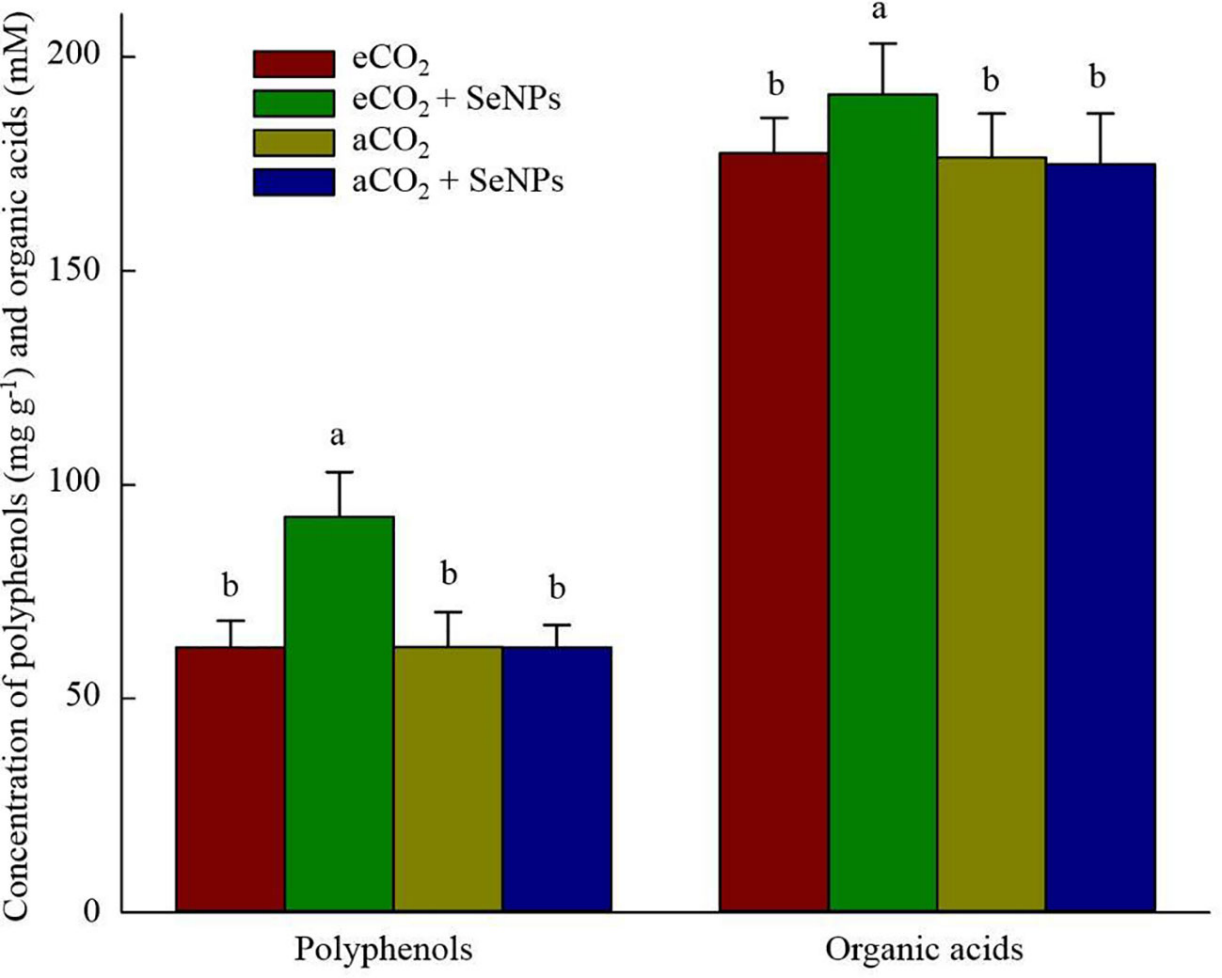
The effect of selenium nanoparticles (SeNPs) on the concentration of organic acids and polyphenols in soil under ambient CO_2_ (aCO_2_) and elevated CO_2_ (eCO_2_). Means in each parameter followed by similar letter(s) are not significantly different at 5% probability level (Tukey test).

The beneficence of SeNPs+eCO_2_ treatment in improving the content of total antioxidant capacity, polyphenols, flavonoids, and total tocopherols (including α-, β-, γ-, and σ-tocopherols) in plants was recorded as equal to 171.3 µmol Trolox g^–1^, 7.6 µg g^–1^, 3.5 µg g^–1^, and 8.4 ng g^–1^, which were about 83, 122, 110, and 102% higher than those in control eCO_2_-treated plants (**Figure 3**). Similarly, the highest concentrations of polyphenols (92.5 mg g^–1^) and organic acids (191.3 mM) in soil were recorded in SeNPs+eCO_2_ treatment, which were significantly higher than other treatments (*P* < 0.05) (**Figure 4**).

### 3.3 Nutrient concentrations in plant

**Figure 5:**
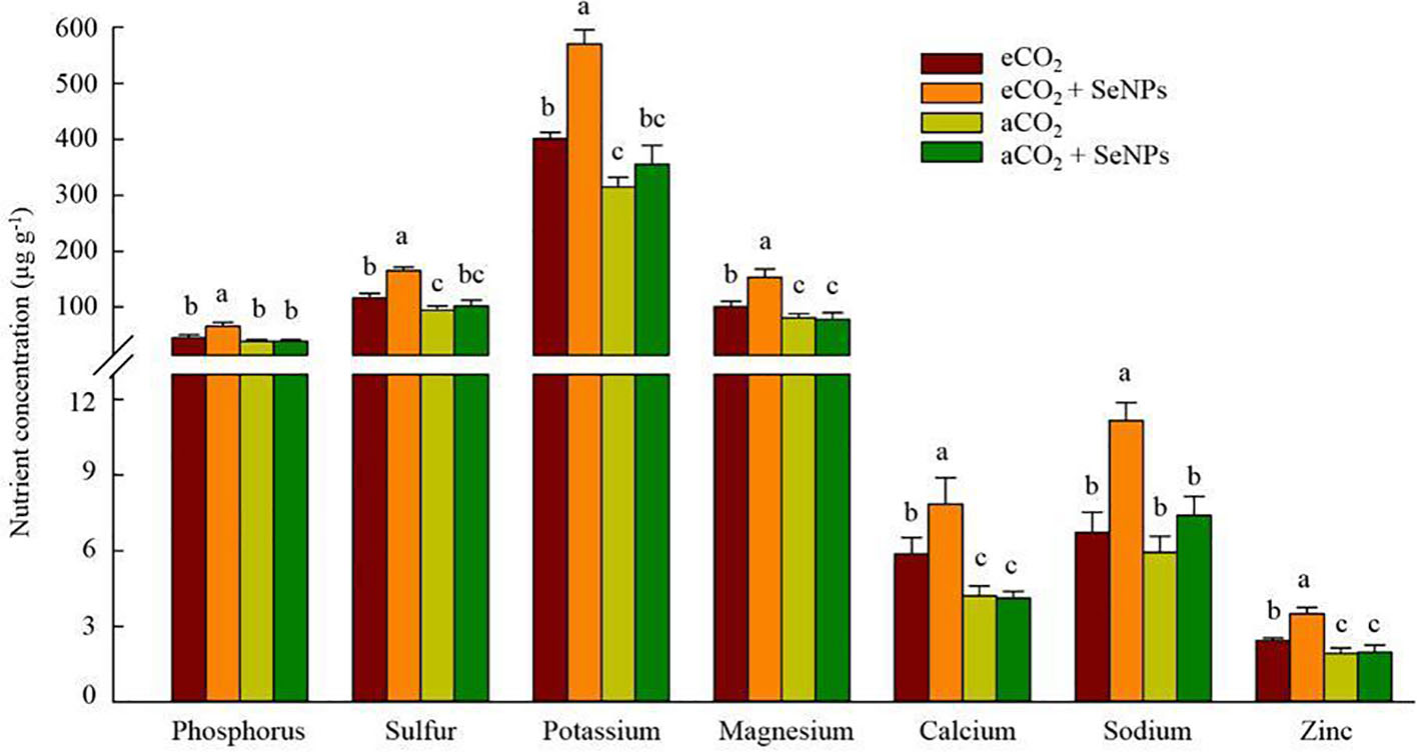
The effect of selenium nanoparticles (SeNPs) on the nutrient concentrations in plant under ambient CO_2_ (aCO_2_) and elevated CO_2_ (eCO_2_). Means in each parameter followed by similar letter(s) are not significantly different at 5% probability level (Tukey test).

The nutrient status of the shoot samples was determined in order to assess the effectiveness of the selenium nanoparticle treatment and its interaction with CO_2_ levels. Accordingly, the concentration of sulfur, potassium, magnesium, calcium, and zinc in shoots was positively affected under elevated CO_2_ treatment, which was more pronounced when SeNPs was applied (+ 34-53%) compared to control eCO_2_-treated plants (**Figure 5**). The results were slightly different for phosphorus and sodium contents which were significantly higher only under the combined treatment (SeNPs+eCO_2_) as compared to other treatments, equal to +47-70% and +51-88%, respectively (**Figure 5**). Interestingly, the individual application of SeNPs did not cause a significant increase (*P* > 0.05) in the concentration of the studied nutrient in plants (**Figure 5**).

### 3.4 Biomass production and selenium content in plant and soil

**Figure 6:**
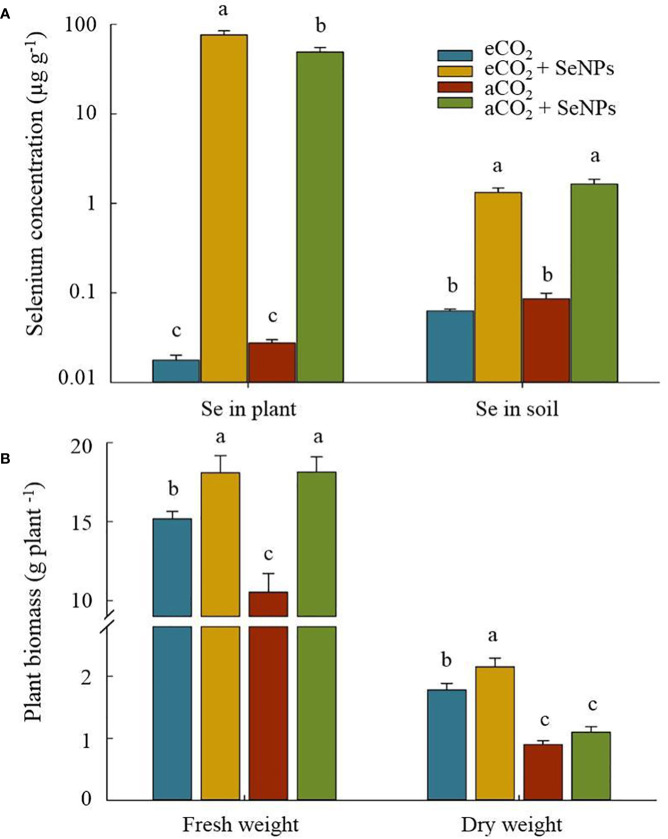
The effect of selenium nanoparticles (SeNPs) on the selenium concentration **(A)** and plant biomass **(B)** under ambient CO_2_ (aCO_2_) and elevated CO_2_ (eCO_2_). Means in each parameter followed by similar letter(s) are not significantly different at 5% probability level (Tukey test).

Although elevated CO_2_ did not affect the accumulation of selenium in soil and plant shoot tissues, the application of of SeNPs significantly enriched the concentration of Se in soil and plants (**Figure 6A**). This remarkable increment in soils treated by SeNPs under eCO_2_ and aCO_2_ conditions were about 4318 and 1783 times higher than that of the control plants at the same CO_2_ level, respectively (**Figure 6A**). The increase in soil Se content resulted in a significant enhancement in the Se accumulation in the plant (*P* < 0.05), which in SeNPs+eCO_2_ and SeNPs+aCO_2_ were 21 and 19 times more concentrated than eCO_2_ and eCO_2_ conditions, respectively (**Figure 6A**). A higher fresh weight belonged to the SeNPs-containing treatments, including SeNPs+eCO_2_ and SeNPs+aCO_2_ which were 20% and 72% higher than the eCO_2_ and aCO_2_ treatments, respectively (**Figure 6B**). This is while the highest dry weight was recorded from eCO_2_-containing treatments, SeNPs+eCO_2_ (2.15 g plant^–1^) and eCO_2_ (1.78 g plant^–1^), which were about +80% and +49% higher than that in control plants under aCO_2_ treatment, respectively (**Figure 6B**).

### 3.5 Correlation analysis

**Figure 7:**
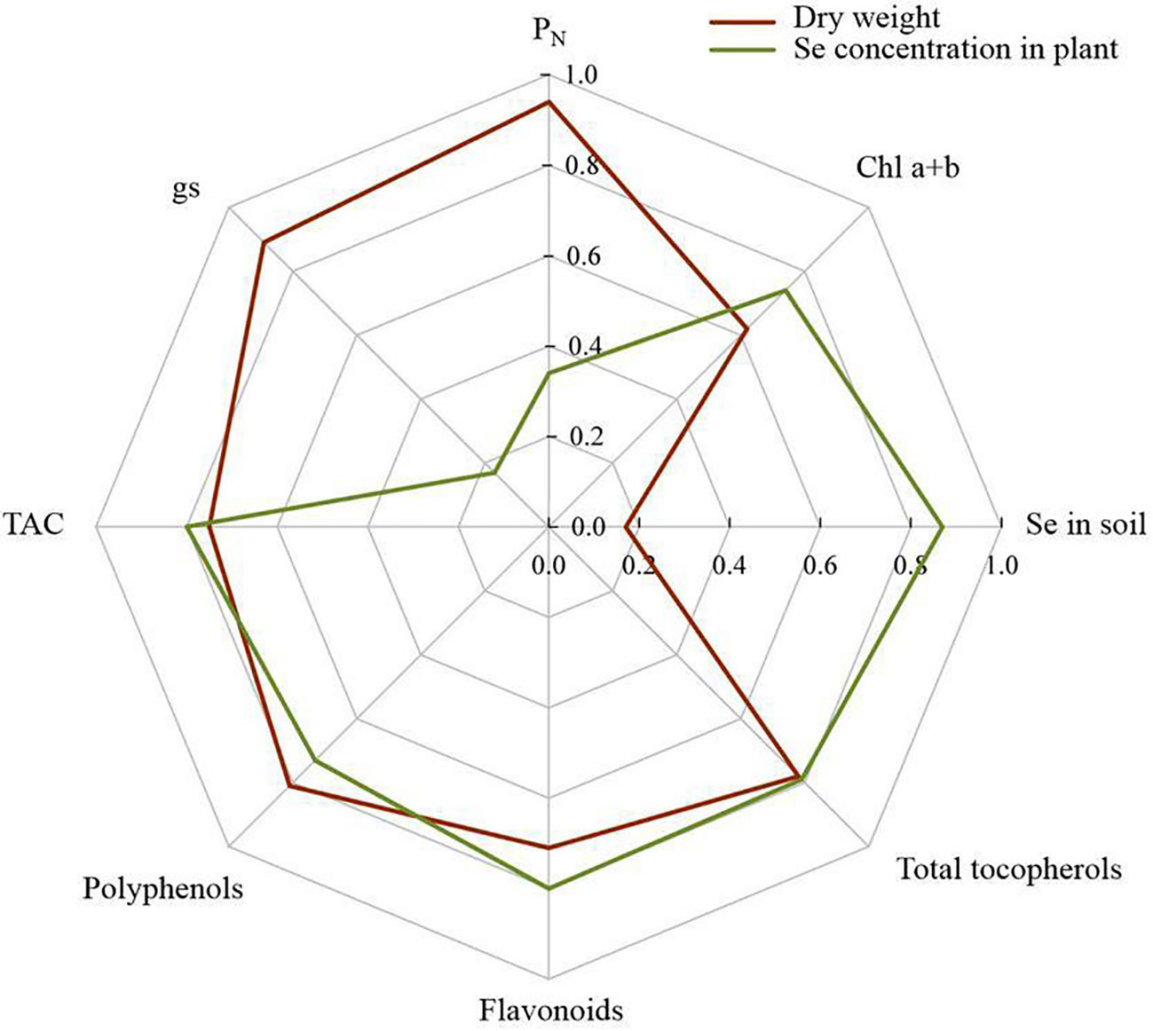
Pearson’s pair-wise correlation coefficients between plant dry matter and selenium content in pant and some other studied variables in this study.

**Figure 7** provides the Pearson correlation analysis relationship between plant biomass, selenium concentration in plants and some key studied traits. In this regard, plant dry weight had a positive correlation with Chl a+b (*r* = 0.62; *P* < 0.05), P_N_ (*r* = 0.94; *P* < 0.01), gs (*r* = 0.89; *P* < 0.01), TAC (*r* = 0.75; *P* < 0.01), polyphenols (*r* = 0.81; *P* < 0.01), flavonoids (*r* = 0.71; *P* < 0.01) and total tocopherols (*r* = 0.81; *P* < 0.01). In contrast, there was no significant relationship between plant dry weight and Se concentration in soil (*r* = 0.17; *P* > 0.05). Also, we didn’t find a correlation between Se concentration in shoot tissues and photosynthesis parameters, including P_N_ and gs (*P* > 0.05). Nevertheless, the Se concentration in the plant not only was correlated to soil Se content (*r* = 0.87; *P* < 0.01), but also had a positive relationship to Chl a+b (*r* = 0.74; *P* < 0.05), TAC (*r* = 0.80; *P* < 0.01), polyphenols (*r* = 0.73; *P* < 0.01), flavonoids (*r* = 0.80; *P* < 0.01) and total tocopherols (*r* = 0.79; *P* < 0.01) (**Figure 7**).

## 4 Discussion

The present study attempted to unravel some of the unanswered questions surrounding the impacts of climate change on crops, one of which is the increase in the atmospheric CO_2_ level. Accordingly, the wheat plant’s responses to the application of selenium nanoparticles were investigated to show whether they can act synergistically and more effectively with elevated CO_2_ on some of the plant’s physiological and biochemical characteristics. eCO_2_ was already found to be influencing wheat physiological and agronomic traits and has been explored in several studies as a factor enhancing wheat biomass and improving net CO_2_ assimilation rate (Broberg et al., 2019; Wang and Liu, 2021). Moreover, agronomic factors (e.g. nutrient supply) were suggested to modulate the eCO_2_ effects on wheat responses (Wang and Liu, 2021). Therefore, SeNPs treatment in the present research can act as a factor in optimizing growth conditions to maximize the eCO_2_-induced benefit in crop production systems.

In order to clarify the mechanisms employed by plants, we first discussed the reactions of photosynthetic pigments and parameters. As shown in **Figure 1**, both SeNPs and eCO_2_ increased the concentrations of Chl a, Chl b, -carotene, lutein, and zeaxanthin, with the combined treatment (SeNPs+eCO_2_) having a greater effect. Although no single study exists that investigates the photosynthesis process in response to SeNPs in eCO_2_-treated plants, extensive research has been carried out on the effects of the individual application of eCO_2_ or selenium biofortification on crops. In this regard, it has already been reported that the protection of chlorophyll structure and function from disruption and degradation was due to the rising concentration of photosynthetic pigments induced by the application of SeNPs (Lanza and dos Reis, 2021; Albqmi et al., 2023) or eCO_2_ conditions (Habeeb et al., 2020) under both stress and non-stress conditions. These findings are relevant to reveal plant status under eCO_2_ once the photochemical reactions are saturated and disperse the excess excitation energy deactivating singlet molecular oxygen (Approbato et al., 2023). eCO_2_ can stimulate a boost in pigment content, mirroring the improved size of the PSII light-harvesting complex by transferring excitation energy favoring the PSII activity (Wang et al., 2015).

Similarly, Silva et al. (2020) considered the improvement of chlorophyll content as one of the main factors for better regulation of the antenna complex, which is associated with an increase in carotenoid content. Lanza and dos Reis (2021) proved that the biosynthesis of such metabolites (e.g., carotenoids, xanthophylls (lutein and zeaxanthin), and β-carotene) in SeNPs-treated plants can improve the energy efficiency of photosynthesis for the better functioning of the photosynthetic system. Since one of the effects of elevated CO_2_ is a deficiency in supplied energy from the light reactions centers, which leads to a decrease in ribulose-1,5-bisphosphate carboxylase/oxygenase (Rubisco) enzyme activity (Jensen, 2000), the possible improvement in the energy efficiency of photosynthesis in SeNPs-treated plants can be one of the explanations for the increase in Rubisco activity at least under eCO_2_ conditions (**Figure 1B**). Nevertheless, improvements in photosynthetic pigments induced by SeNPs could not result in further improvements in the photochemistry of photosynthesis under ambient CO_2_ conditions, despite the obvious amelioration that was recorded for photosynthesis rate (P_N_) and stomatal conductance (gs) in eCO_2_-treated plants (**Figure 1B**). These results showed that the boost in photosynthetic capacity in eCO_2_-treated plants in our research was more related to the enhancement of Rubisco enzyme activity than to photosynthetic pigment content, especially since Rubisco activation is the principal limitation to CO_2_ fixation during photosynthesis under elevated CO_2_ conditions (Jensen, 2000). Also, it has already been reported that increases in the stomatal conductance in plants are associated with more activation of Rubisco, as one of the non-stomatal and active parameters in the Calvin cycle (Acosta-Motos et al., 2017; Yaghoubi Khanghahi et al., 2021) which in turn is one of the reasons for improving the photosynthetic capacity under eCO_2_ in the present study. On the other hand, Fv/Fm ratios in the current research were 0.79-0.80 and were not affected by the studied treatments (**Figure 1B**). It has previously been proven that this parameter, as the expression of the maximum efficiency of PSII in dark-adapted leaves, usually ranges from 0.79 to 0.85 under non-stress conditions (Yaghoubi Khanghahi et al., 2019). Similar to our results, recent evidence suggests that the Fv/Fm ratio is less sensitive to non-stress conditions or even mild environmental stress and can remain unchanged (Yaghoubi Khanghahi et al., 2021).

Similar to photosynthetic, the combined treatment (SeNPs + eCO_2_) had the highest content of antioxidant enzymes and metabolites (**Figures 2**, **3**), which are known for their involvement in direct or indirect reactive oxygen species (ROS) scavenging (Albqmi et al., 2023). In accordance with the present results (except for POX), previous studies have demonstrated no significant increase in antioxidant enzymes and metabolites in control plants under eCO_2_ compared to aCO_2_ conditions (Mishra et al., 2013; AbdElgawad et al., 2016) and linked it to the lack of a significant increase in ROS content (AbdElgawad et al., 2016). Although the stress biomarkers that could confirm oxidative stress were not investigated in the current study, it seems that there was no increase in the ROS content in eCO_2_-treated control plants. If we accept this explanation, then the sharp increase in the content of antioxidant enzymes (POX, CAT, SOD, APX, DHAR, MDHAR, GR, and GPX) and metabolites (ASC, GSH, TAC, polyphenols, flavonoids, and tocopherols) in SeNPs-treated plants under eCO_2_ conditions (**Figures 2**, **3**) should be interpreted with caution. It is difficult to explain this result, but it might be related to nearly 50% increase in sodium concentration in the combined (SeNPs+eCO_2_) treatment compared to other treatments (**Figure 5**). So, this response may explain the relatively greater content of antioxidant components in plants treated by SeNPs under eCO_2_. The increase in Na concentration in shoots did not have an antagonistic relationship with the uptake of ions K^+^ and Ca^2+^ (Yu and Assmann, 2016), because the concentration of K and Ca was also increased in SeNPs-treated plants under eCO_2_ (**Figure 5**); therefore it seemingly independently caused oxidative stress in these plants (Lanza and dos Reis, 2021). It has already been proposed that Se can individually enter the root system via passive diffusion (through the aquaporin membrane channels) to be rapidly assimilated as selenite and/or organic Se, followed by transportation to the shoot (Lyu et al., 2022), and consequently, Se in the plant has been suggested to have dual impacts on the nutrient uptake by plants, not merely inhibitory impacts (Gui et al., 2022). It was suggested that Se ions can also change the permeability coefficients of some ions (e.g. Na^+^) in biomembranes and influencing the transportation of those ions via the membrane (Kaur et al., 2014; Bano et al., 2021). Therefore, a 50% increase in Na concentration in SeNPs+eCO_2_ treatment may be explained by the fact that it is the quantitative content of Se and essential nutrients in plants that can specify the content of Na and the interaction between Se and Na in plants, resulting in antagonistic and/or synergistic effects (Bano et al., 2021).

Elkelish et al. (2019) considered the stimulation of such enzymatic and non-enzymatic antioxidant activity in plant cells as a mechanism for transporting Na^+^ to the vacuoles to prevent the adverse effects of excess Na-induced damage. Lanza and dos Reis (2021) and Habibi (2017) also reported the positive effects of Se through compartmentalizing Na^+^ in vacuoles and increasing the bond with the cell wall in high Na-containing plants. Better nutrient absorption (**Figure 5**) and higher biomass production by the plants in SeNPs+eCO_2_ treatment (**Figure 6B**) can indicate the high efficiency of the plant’s defense system, which indeed prevents the disruption of the plants’ mineral balance and various physiological processes under high Na content (Bano et al., 2021). Moreover, the higher uptake of minerals (**Figure 5**) and dry matter production (**Figure 6B**) in SeNPs+eCO_2_ treatment in our study mirror those of the previous research, which reported an improved photosynthesis rate and increased photosynthates allocation to the roots in response to eCO_2_ and SeNPs (Habeeb et al., 2020; Albqmi et al., 2023), which in turn, resulted in strengthening the capacity of roots to uptake more nutrients from the soil and produce biomass (Albqmi et al., 2023; Du et al., 2023).

There was a clear increase in the accumulation of selenium in rhizospheric soil and plant shoot tissues in response to SeNPs application, a result that was not recorded in eCO_2_-treated plants (**Figure 6A**). Nonetheless, the results of the correlational analysis didn’t show a significant correlation between Se concentration and dry matter production in the plant (**Figure 7**), one of the reasons for which might be the lack of significant effect of SeNPs on the photochemical parameters of the photosynthesis process, such as Rubisco activity, stomatal conductance and photosynthetic capacity (**Figure 1B**). Consequently, elevated CO_2_ could be a principal factor, if not the only one, causing the positive changes in photosynthetic sink-source balance in plants by overcoming one of the main limitations of photosynthesis (Jensen, 2000), which in turn yielded a contribution to plant growth and biomass production (Yaghoubi Khanghahi et al., 2021).

The current study is based on a recommended dose of 25 mg L^–1^ SeNPs (Albqmi et al., 2023) and the consequent Se content in plant tissues (49-76 µg g^–1^) and rhizospheric soil (1.3-1.6 µg g^–1^) in SeNPs-treated plants was in the normal range that was previously reported (0.005-5500 µg g^–1^ for plants and 0.02-100 µg g^–1^ for soil) to be safe for plants and humans (Gupta and Gupta, 2000; Lanza and dos Reis, 2021). Nevertheless, considering the intense increase in Se content in rhizospheric soil (19-21 times higher than control) and plant (1783-4318 times higher than control), our results may not be applicable to the long-term application of SeNPs, especially under elevated CO_2_. In other words, an important unanswered question that arises here is whether the application of SeNPs in the long term can break the border of toxic and beneficial effects of Se on plants, especially in the soil that will face the gradual accumulation of Se over time. Moreover, although, there has been considerable discussion about the low risks of the SeNPs in the agricultural section so far (Morales-Espinoza et al., 2019; Lanza and dos Reis, 2021), it seems that caution should be applied to extrapolate those previous findings to plants grown under elevated CO_2_ conditions since the Na was significantly absorbed by SeNPs-treated plants in our study. So, it seems that further research is needed to propose optimal doses of SeNPs, in particular under elevated CO_2_ conditions.

## 5 Conclusion

The obtained results showed that the synergistic effects of selenium nanoparticles and elevated CO_2_ conditions are better than those of only eCO_2_. The benefits of SeNPs+eCO_2_ treatment in improving the content of total antioxidant capacity, polyphenols, flavonoids, and total tocopherols in plants were recorded. SeNPs+eCO_2_ treatment resulted in the highest accumulation of photosynthetic pigment content in leaves. Under eCO_2_ only, positive impacts on Rubisco activity and stomatal conductance were observed. Further research is needed to propose optimal doses of SeNPs, in particular under elevated CO_2_ conditions.

## Data availability statement

The raw data supporting the conclusions of this article will be made available by the authors, without undue reservation.

## Author contributions

All authors have contributed equally to the research and analysis of the various results sections within the review. All have corrected and modified the different versions of the manuscript as prepared by the corresponding and senior authors. All authors contributed to the article and approved the submitted version.
